# Disparities in Health Care Delivery and Hospital Outcomes between Non-Saudis and Saudi Nationals Presenting with Acute Coronary Syndromes in Saudi Arabia

**DOI:** 10.1371/journal.pone.0124012

**Published:** 2015-04-16

**Authors:** Hussam F. AlFaleh, Mostafa Q. Al Shamiri, Anhar Ullah, Khalid F AlHabib, Ahmad Salah Hersi, Shukri AlSaif, Khalid AlNemer, Amir Taraben, Asif Malik, Ahmed M Abuosa, Mimish LA, Tarek Kashour

**Affiliations:** 1 Cardiac Sciences department, College of Medicine, King Saud University, Riyadh, Saudi Arabia; 2 Saud AlBabtain Cardiac Center, Dammam, Saudi Arabia; 3 Medicine Department, Security forces Hospital, Riyadh, Saudi Arabia; 4 King Faisal Specialist Hospital and Research Center, Jeddah, Saudi Arabia; 5 King Fahad General Hospital, Jeddah, Saudi Arabia; 6 King Khalid National Guard Hospital, Jeddah, Saudi Arabia; 7 King Abdul Aziz University Hospital, King Abdulaziz University, Jeddah, Saudi Arabia; University Heart Center Freiburg, GERMANY

## Abstract

**Background:**

Saudi Arabia has a non-Saudi workers population. We investigated the differences and similarities of expatriate non-Saudi patients (NS) and Saudi nationals (SN) presenting with acute coronary syndromes (ACS) with respect to therapies and clinical outcomes.

**Methods:**

The study evaluated 2031 of the 5055 ACS patients enrolled in the Saudi Project for Assessment of Acute Coronary Syndrome (SPACE) from 2005 to 2007. Propensity score matching and logistic regression analysis were performed to account for major imbalances in age and sex in the two groups.

**Results:**

The mean patient age was 56.2±9.8, and 83.5% of the study cohort were male. SN were more likely to have risk factors of atherosclerosis. ST-elevation MI (STEMI) was the most common ACS presentation in NS, while non-ST ACS was more common in SN. The median symptom-to-door time was significantly greater in NS patients (Median 175 min (197) vs. 130 min (167), p=0.027). The only difference in pharmacological therapies between the two groups was that NS were more likely to receive fibrinolytic therapy. NS were less likely than SN to undergo percutaneous coronary interventions (PCI; 32.6% vs. 42.8%, p=0.0001) or primary PCI (7.8% vs. 22.8%, p<0.001). Hospital mortality, cardiogenic shock, and heart failure were significantly higher in NS compared to SN. After adjusting for baseline variables and therapies, the odds ratios for hospital mortality and cardiogenic shock in NS were 2.9 (95% CI 1.5–6.2, p=0.004) and 2.8 (95% CI 1.5–4.9, p<0.001), respectively.

**Conclusion:**

Our findings indicate disparities in hospital care between NS and SN ACS patients. NS patients had worse hospital outcomes, which may reflect unequal health coverage and access-to-care issues.

## Introduction

Acute coronary syndrome (ACS) is a growing public health problem in the Middle East and poses an economic burden [1,2]. Like many Arab gulf countries, Saudi Arabia has a large guest worker non-Saudi work force. In the last Saudi national census in 2010, the expatriate population comprised at least 30% of the general population of Saudi Arabia [3] The non-Saudi population has distinct racial, socioeconomic, and demographic characteristics; accordingly, the received health care, response to therapy, and clinical outcomes may differ in this population compared to the population of Saudi nationals.

The Saudi Project for Assessment of Coronary Events (SPACE) registry is the first national study to provide a comprehensive overview of current diagnostic and treatment strategies for ACS patients in Saudi Arabia [4,5]. Our objectives in this study were to investigate the clinical presentation, hospital care and treatment strategies, and hospital outcomes in non-Saudi expatriate ACS patients and to compare them with those of Saudi national ACS patients based on data from the SPACE registry.

## Methods

The SPACE study was the first prospective, multicenter, observational study of all consecutive ACS patients admitted to participating hospitals across Saudi Arabia. The objectives of SPACE were to study current practice patterns in the management of ACS, assess the gap between clinical practice and guidelines, and potentially improve the quality of cardiac care. The SPACE study was conducted from 1 December, 2005 to 31 December, 2007. The full description of the methods was published previously [4,5]. Briefly, 17 urban hospitals in 7 cities that were representative of 5 regions in Saudi Arabia participated in the SPACE registry. Of the participating hospitals, 12 (70%) had a cardiac catheterization lab, while just 2 (12%) of the hospitals offered around the clock primary percutaneous coronary interventions (PCI) for all of their ST-elevation myocardial infarction (STEMI) patients.

To avoid double-counting patients, each patient’s national identification number was used, however no other identifiers such as patient’s name were included in the Case Report Form (CRF), and the identity of patients was anonymized all throughout the process of data analysis. The following data were collected using the CRF: demographic information; past medical history; provisional diagnosis on admission and final discharge diagnosis; ECG findings; laboratory investigations; medical therapy used on admission, during hospitalization, and on discharge; use of cardiac procedures and interventions; adverse in-hospital outcomes; and in-hospital mortality. The different types of ACS were categorized based on the definitions of the Joint Committee of the European Society of Cardiology/American College of Cardiology (ACC) [6].

Given that our initial analysis of the SPACE data showed that the non-Saudi cohort was predominantly male and that the median age were significantly lower than that of the Saudi cohort, we performed propensity score matching to account for these two major demographic imbalances. Matching was also performed because our primary objective was to assess process of care and the resulting outcomes given similar circumstances. Baseline characteristics, hospital therapies, and clinical adverse outcomes were compared between Saudi nationals and non-Saudis. Ethics approval was obtained from the institutional review board (IRB) of King Khalid university Hospital, King Saud University (The principle coordinating center), as well as from all participating hospitals (S1 Hospitals List). Given that the SPACE registry is in part a quality improvement initiative, and that patients identity were anonymized to the analyzers, the IRB did not require a written informed consent.

### Statistical analysis

Categorical data were reported as absolute numbers and percentages. Numeric data were reported as means and standard deviations (SD) or as median values and interquartile range. The propensity score was calculated using logistic regression modeling with ‘sex’ and ‘age’ as covariates. For each non-Saudi, 3 age- and sex- matched propensity scores from Saudi nationals were identified using the SAS ‘1: N Matching’ macro. Comparisons between groups were performed using the chi-square test for categorical variables. We used the independent sample t-test or Mann-Whitney U test for continuous variables for unmatched data. We used McNemar's test for categorical variables and paired samples t-tests or the Wilcoxon signed rank sum test for continuous variables for matched data. The adjusted odds ratios were calculated using conditional logistic regression models adjusted for a history of hypertension, hyperlipidemia, peripheral artery disease (PAD), coronary artery bypass graft surgery (CABG), diabetes mellitus, heart failure, percutaneous coronary intervention (PCI), and discharge diagnosis. Some of the risk factors were not included in the model due to missing data, despite significant differences between the two groups (i.e. Saudi nationals versus non-Saudis). All analyses were performed using SAS/STAT software, Version 9.2 (SAS Institute Inc., Cary, NC, USA).

## Results

A total of 5055 ACS patients from 17 hospitals enrolled in the SPACE registry. Prior to age and gender matching, Saudi nationals were significantly older than non-Saudis (59.7±12.8 years vs. 50.2±10.5 years, *p*<0.0001), and there were significantly more males in the non-Saudi group (91.3% vs. 74.5%, *p*<0.0001), (S1, S2, and S3 Tables). Of the 5055 patients, 2031 patients were matched according to age and sex. Table 1 shows the baseline characteristics of the study cohort. The mean age of the study population was 56.2 ± 9.8 years, and the vast majority were male (83.5%). Compared to non-Saudi patients, Saudis had significantly higher rates of diabetes (60% vs. 50.1%, *p*<0.0001), hypertension (55.1% vs. 48%, *p* = 0.01), and hyperlipidemia (48% vs. 42.1%, *p* = 0.03). More non-Saudis than Saudis had a past history of CABG (5.7% vs. 5.9%, *p*<0.0001) and PAD (5.9% vs. 8.7, *p* = 0.038). On the other hand, Saudi patients were more likely to have a history of PCI (15.4% vs. 11.2%, *p* = 0.0003) and cerebrovascular disease compared to non-Saudis (4.8% vs. 2.8%, *p*<0.0001). With regard to the type of ACS presentation, Saudi patients were more likely to present with unstable angina or non-ST elevation myocardial infarction (NSTEMI), while non-Saudis were more likely to present with STEMI. Moreover, non-Saudi patients were more likely to present with symptoms of heart failure (22.8% vs. 17.7%, *p* = 0.017). In patients presenting with STEMI, the median time from the onset of symptoms until hospital arrival was significantly longer in non-Saudis compared to Saudis [Median 130 min (167) vs. 175 min (197), *p* = 0.027].

**Table 1:** Baseline characteristics of study patients with acute coronary syndrome.

	All patients n = 2031	Saudi nationals n = 1523	Non-Saudis n = 508	*p*-value
Age, y, mean ± SD	56.15 ± 9.77	56.19 ± 9.81	56.03 ± 9.66	0.7511
Male, n (%)	1695 (83.46)	1263 (82.93)	432 (85.04)	0.3010
Diabetes mellitus, n (%)	1181 (58.26)	927 (60.99)	254 (50.10)	<0.0001
Hypertension, n (%)	1079 (53.31)	836 (55.11)	243 (47.93)	0.0144
Hyperlipidemia, n (%)	862 (46.49)	668 (47.95)	194 (42.08)	0.0312
Smoking, n (%)	891 (44.15)	667 (44.03)	224 (44.53)	0.1982
CAD, n (%)	282 (13.92)	204 (13.47)	78 (15.38)	0.3081
Past PCI, n (%)	293(14.4)	236 (15.4)	57 (11.2)	0.0003
CABG, n (%)	116 (5.72)	86 (5.65)	30 (5.91)	<0.0001
CVA, n (%)	87 (4.29)	73 (4.81)	14 (2.76)	<0.0001
PAD, n (%)	134 (6.62)	90 (5.94)	44 (8.66)	0.0388
Unstable angina, n (%)	472 (23.24)	374 (24.56)	98 (19.29)	0.0006
STEMI, n (%)	873 (42.98)	618 (40.58)	255 (50.20)	
NSTEMI, n (%)	686 (33.78)	531 (34.87)	155 (30.51)	
Heart failure, n (%)	346 (19.04)	237 (17.71)	109 (22.76)	0.0176
Symptom-to-hospital arrival time, minutes, median (IQR) ^a^	148.5 (180.5)	130.0 (167.0)	175.0 (197.0)	0.027
HR < 100, n (%)	1562 (85.64)	1156 (86.01)	406 (84.58)	0.4489
SBP ≤ 90, n (%)	63 (3.46)	43 (3.20)	20 (4.18)	0.3110
Waist circumference, n (%)	100.9 ± 17.92	100.3 ± 17.41	102.2 ± 18.90	0.2417
Body mass index, n (%)	28.29 ± 5.26	28.44 ± 5.25	27.90 ± 5.26	0.0901
Total cholesterol, mean ± SD	4.55 ± 1.31	4.47 ± 1.25	4.80 ± 1.44	<0.0001
LDL cholesterol, mean ± SD	2.82 ± 1.53	2.79 ± 1.62	2.94 ± 1.16	0.1806
FBS, mean ± SD	7.76 ± 3.12	7.74 ± 3.12	7.81 ± 3.11	0.7038
Hemoglobin, mean ±SD	13.98 ± 2.02	13.92 ± 2.03	14.17 ± 1.98	0.0161
Creatinine, mean ± SD	103.9 ± 66.99	103.2 ± 68.00	106.2 ± 63.92	0.3857
eGFR mean ± SD (ml/min/1.73 m²)	77.01 ± 23.51	77.61 ± 23.43	75.24 ± 23.68	0.051
LV EF (< 35%), n (%)	669 (35.21)	513 (35.92)	156 (33.05)	0.2666
Coronary angiography, n (%)	1409 (96.37)	1113 (73.1)	296 (58.3)	<0.0001
LMS disease, n (%)	61 (4.33)	51 (4.59)	10 (3.38)	0.4245
3-vessel disease, n (%)	470 (33.38)	358 (32.19)	112 (37.84)	0.0715
3-vessel or LM disease, n (%)	500 (35.51)	383 (34.44)	117 (39.53)	0.1159

CABG, coronary artery bypass surgery; CAD, coronary artery disease; CVA, cerebrovascular accidents; eGFR, estimated glomerular filtration rate; FBS, fasting blood sugar; HR, heart rate; IQR, inter-quartile range; LMS, left main stem; LVEF, left ventricular ejection fraction; NSTEMI, non-ST elevation myocardial infarction; PAD, peripheral arterial disease; PCI, percutaneous coronary intervention; SBP, systolic blood pressure; SD, standard deviation; STEMI, ST elevation myocardial infarction.

^a^ Data from all STEMI patients.

With respect to baseline blood findings, non-Saudis were more likely to be anemic and to have higher levels of total cholesterol compared to Saudis, though the mean LDL cholesterol levels were not different. There were no differences between the two groups in the frequency of significant left ventricular systolic dysfunction, which was defined as an ejection fraction <35%. Saudis were more likely than non-Saudis to undergo diagnostic coronary angiograph; however, there were no differences between the two groups with respect to the rates of high-risk coronary anatomy (Table 1).

### Hospital therapies

Significant differences were observed in medical therapy prior to hospitalization between Saudis and non-Saudis (Table 2). With the exception to fibrinolytic therapy, there were no significant differences in the hospital medical therapies received by Saudis versus non-Saudis, and both groups received high rates of evidence-based medicines (Table 2). In STEMI patients, fibrinolytic therapy was used more frequently in non-Saudis compared to Saudis (82.4% vs. 65%, p<0.001), and when used, the median door-to-needle time was shorter in non-saudis [45 min (45) vs. 57 min (74), *p* = 0.007]. Although the rate of primary PCI in the entire study cohort was low (16.6%), the rate of primary PCI was significantly higher in Saudis compared to non-Saudis (12.8% vs. 7.8%, *p*<0.001). Even though primary PCI was used less frequently in non-Saudis, the median door-to-balloon time was shorter in these patients compared to Saudis [85.5 min (88) vs. 112.5 min (14.5), p = 0.045]. Moreover, the rate of those with a door-to-balloon time < 90 minutes was significantly higher in non-Saudis (Table 2). PCI rates in general (determined either as primary or after the index event) were higher amongst Saudi patients, while rates of CABG were not different between the two groups (Table 3).

**Table 2:** Management of study patients with acute coronary syndromes.

	All patients n = 2031	Saudi nationals n = 1523	Non-Saudis n = 508	*p*-value
**Medical therapy prior to hospitalization**				
Aspirin, n (%)	888 (62.54%)	705 (65.34%)	183 (53.67%)	<0.001
Clopidogrel, n (%)	373 (26.25%)	295 (27.34%)	78 (22.81%)	0.105
Beta Blockers, n (%)	694 (48.91%)	533 (49.44%)	161 (47.21%)	0.494
ACEI, n (%)	557 (39.20%)	443 (41.06%)	114 (33.33%)	0.011
ARBs, n (%)	92 (6.53%)	73 (6.84%)	19 (5.57%)	0.452
Statins n, (%)	751 (52.85%)	611 (56.63%)	140 (40.94%)	<0.001
**Hospital management**				
Aspirin n (%)	1983 (97.88)	1486 (97.83)	497 (98.03)	0.8607
Clopidogrel, n (%)	1683 (83.07)	1274 (83.87)	409 (80.67)	0.1009
β blockers, n (%)	1663 (82.16)	1254 (82.61)	409 (80.83)	0.3834
ACEI, n (%)	1426 (70.38)	1080 (71.10)	346 (68.24)	0.2381
ARBs, n (%)	113 (5.61)	87 (5.77)	26 (5.15)	0.6560
Statins, n (%)	1889 (93.24)	1415 (93.15)	474 (93.49)	0.8387
Fibrinolytic therapy^a^, n (%)	283 (72.38)	147 (65.04)	136 (82.42)	< 0.001
Primary PCI^a^, n (%)	65 (16.55)	52 (22.81)	13 (7.83)	<0.001
Door-to-needle time, min, median (IQR)^a^	50.00 (53.00)	57.00 (74.00)	45(45)	0.007
Door-to-balloon-time, min, median (IQR)^a^	107.5 (78.00)	112.5 (88.00)	85.5(14.50)	0.047
Door-to-needle-time < 30 min, n (%)^a^	60 (21.20)	25 (17.01%)	35 (25.74)	0.082
Door-to-balloon-time < 90 min, n (%)^a^	22 (37.93)	14 (30.43)	8 (66.67)	0.042
PCI, n (%)	742 (40.17)	586 (42.84)	156 (32.57)	0.0001
CABG, n (%)	170 (9.13)	121 (8.76)	49 (10.19)	0.3585
Length of hospital stay, Median (IQR)	4(5)	4(5)	5(5)	0.125

ACEI, angiotensin converting enzyme inhibitors; ARBs, angiotensin receptor blockers; CABG, coronary artery bypass surgery; IQR, inter-quartile range; PCI, percutaneous coronary intervention.

^a^ Data from all STEMI patients.

**Table 3:** Adverse hospital outcomes of study patients with acute coronary syndromes.

Outcome n (%)	All patients n = 2031	Saudi nationals n = 1523	Non-Saudis n = 508	*p*-value	*p*-value^a^
Death	46 (2.26)	25 (1.64)	21 (4.13)	0.003	0.0005
Heart failure	170 (8.37)	115 (7.56)	55 (10.83)	0.021	0.0201
Stroke	14 (0.69)	9 (0.59)	5 (0.98)	0.356	0.2328
Re-infarction	25 (1.23)	19 (1.25)	6 (1.18)	0.906	0.9393
Cardiogenic shock	83 (4.09)	52 (3.41)	31 (6.11)	0.007	0.0036
Major bleeding	18 (0.89)	12 (0.79)	6 (1.18)	0.411	0.3373

^a^Using conditional logistic regression to account for matching.

### Hospital outcomes

Overall mortality rates were significantly higher in non-Saudis (4.1% vs. 1.6%, *p* = 0.0005), as were heart failure rates (10.8% vs. 7.6%, *p* = 0.02) and cardiogenic shock rates (6.1% vs. 3.4%, *p* = 0.003). There were no differences between the two groups with respect to the rates of re-infarction, major bleeding, and stroke (Table 3).

Non-Saudi status was a significant predictor for death, heart failure, and cardiogenic shock (Table 4). After adjusting for the type of ACS presentation and for risk factors, heart failure at presentation, PAD, and revascularization procedures, non-Saudi status remained an independent predictor of mortality [odds ratio 2.9 (1.5–6.2), p = 0.004], and cardiogenic shock [2.8 (1.5–4.9), p<0.001].

**Table 4:** Crude and adjusted odds ratios (ORs) for developing adverse hospital outcomes in non-Saudi patients with acute coronary syndromes.

Outcome	Crude OR (95% CI)	*p*-value	Adjusted OR (95% CI)	*p*-value
Death	2.78 (1.52–5.07)	< 0.001	2.91 (1.45–6.15)	0.004
Heart failure	1.51 (1.06–2.12)	0.020	1.41 (0.86–2.30)	0.168
Stroke	1.95 (0.63–5.99)	0.232	1.66 (0.38–7.23)	0.500
Re-infarction	1.04 (0.41–2.65)	0.939	1.48 (0.48–4.52)	0.490
Cardiogenic shock	1.96 (1.23–3.12)	0.003	2.75 (1.54–4.93)	<0.001
Major bleeding	1.61 (0.60–4.35)	0.337	1.76 (0.55–5.62)	0.337

## Discussion

This is the only study to date to investigate treatment and outcome disparities between Saudi nationals and non-Saudi expatriates that presented with ACS at hospitals in Saudi Arabia. In this sub-study of the SPACE registry, we found several differences between Saudis and non-Saudis who presented with ACS. First, at baseline, non-Saudi patients had fewer risk factors for atherosclerosis and were more likely to have suffered from atherosclerotic disease as evidenced by higher rates of PAD and previous CABG. These non-Saudi patients were more likely to present with STEMI and heart failure, both high-risk ACS presentations. Second, although the overall utilization of evidence-based medicine was not different in the two groups, we found that non-Saudis with STEMI were more likely to receive fibrinolytic therapy and to receive it in a timely manner. The higher utilization of fibrinolytic therapy in non-Saudis might be expected given the high STEMI rates. On the flip side, non-Saudis were less likely to undergo primary PCI, but, interestingly, once PCI was performed, they were more likely to have favorable door-to-balloon time. Lastly, despite favorable reperfusion therapy time indicators, we found that non-Saudis had an almost 3-fold higher risk of mortality and of developing cardiogenic shock compared to Saudi nationals.

It is unclear why non-Saudis are at such a disadvantage with respect to clinical outcomes despite generally receiving adequate evidence-based medical therapies. As noted above, compared to Saudis, non-Saudis more often presented with high-risk features (STEMI and heart failure). This could explain the higher rate of adverse outcomes in this group; however, we found that non-Saudi status was still a predictor of death and cardiogenic shock even after adjusting for these factors in the logistic regression model. One of the central findings of this analysis is that the median chest pain-to-hospital arrival time was 175 min in non-Saudis, which was 45 minutes longer than in Saudis. Although both the median door-to-needle time and the median door-to-balloon time were shorter in non-Saudis, this apparent short door-to-reperfusion time might have been offset by the longer pre-hospital delay in non-Saudis. It is well documented that longer symptom-to-reperfusion time results in higher mortality, regardless of the choice of reperfusion therapy [7]. In addition, the highest myocardial salvage rate is within the first two hours following chest pain onset, and delays in administrating reperfusion therapy is associated with a non-linear increase in mortality [7,8]. The STEMI guidelines’ emphasis on reducing the time from first contact with medical care to reperfusion has reduced hospital delays; however, pre-hospital delays remain a challenge [9,10,11].

The symptom-to-hospital delay documented in non-Saudis may reflect patient characteristics as well as system-related gaps. Substantial segments of the non-Saudi worker population are blue-collar workers who are mainly from south Asia and do not speak Arabic, which is the official language in Saudi Arabia. Thus, communication issues might be a factor in the lack of timely access to proper health care or delays in diagnosis and therapies, although our findings do not necessarily support this premise.

Notably, non-Saudi expatriates are not eligible for free governmental health care and can only be treated in private hospitals. It is conceivable that non-Saudi patients might initially present at a private clinic or a hospital, then decide to go to a governmental hospital in view of the high cost of care. This is most likely if the patients are uninsured or if they are insured with a health policy that does not cover the major expenses associated with cardiac care, including invasive procedures. Over the last 10 years, the government has mandated comprehensive health insurance coverage for the non-Saudi population, which makes up more than 30% of Saudi Arabia’s population according to the most recent census, conducted in 2010 [3]. Private business owners are required to pay for health insurance coverage; however health insurance coverage and quality is variable in Saudi Arabia. A system of multi-payer health insurance is currently used for the medical treatment of the expatriate population in Saudi Arabia. Within this system, insurance companies negotiate health packages with health providers that ensure profit for their companies. This type of health coverage, and differences in premiums, might result in inequalities in the services provided to patients [12]. Such premium-based variability in insurance coverage could restrict patients’ eligibility for coverage at certain hospitals, and some hospitals might not offer state-of-the-art cardiac care, such as coronary angiography. This could explain, at least in part, why non-Saudis are less likely to undergo diagnostic coronary angiography and PCI.

Socio-economic level has been shown to predict referral to procedures such as cardiac catheterization after myocardial infarction as well as long-term outcomes, even in the presence of universal health coverage and in other contexts [13,14]. Furthermore, studies conducted in the United States have shown a strong interaction between race and the likelihood that PCI is performed [15]. As noted, substantial sectors of the non-Saudi worker population are blue-collar and domestic workers who are in a low-income bracket.

Inherent physician bias might theoretically be a factor in the observed differences in outcomes between Saudis and non-Saudis, but our data did not show this. On the contrary, our analysis showed that non-Saudis had a more favorable door-to-reperfusion time.

This study has several limitations. Our data were based on an observational study, and this type of study has inherent biases that cannot be avoided. Unfortunately, the involvement of private hospitals was limited in the SPACE registry, and therefore we could not adequately investigate the pattern of care and outcomes in these institutions and compare them with government hospitals. Further, it is conceivable that hospitals that participated in the SPACE registry are generally motivated and have more resources. Notwithstanding these limitations in hospital representation, the disparities might have been more striking if private hospitals had been included because of the gaps in resources between government and private hospitals. The non-Saudi population is relatively young and mostly male, findings that were confirmed in the SPACE registry. Data collection was not detailed enough to address questions of race or socio-economical status, and therefore we cannot investigate the interaction of these two factors with process of care and outcomes. We cannot account for unmeasured variables that might have played a role in patients’ adverse outcomes. These data reflect the situation during the study period i.e. in 2005–2007, and some factors may have changed in subsequent years. However, these data are still relevant as baseline data and, therefore, additional studies are needed to determine whether changes have occurred. Lastly, we did not collect post-discharge data on adherence to medical therapy, follow-up, or long-term outcomes, which may be considered a limitation of the study.

## Conclusions

Here we found disparities in the clinical features, use of invasive procedures, and adverse outcomes between Saudi nationals and non-Saudi guest workers presenting with ACS at hospitals in Saudi Arabia. Policy makers need to take these disparities into account and to address systematic barriers to timely ACS care with a particular emphasis on equitable health care coverage. Comprehensive, equitable health coverage should address primary as well as secondary prevention. Further research is needed to investigate the causes underlying the pre-hospital delays in presentation, the disparities in performing invasive procedures, the discharge and post-hospital care, and long-term outcomes in non-Saudis.

## Supporting Information

S1 Hospitals List List of Ethics boards approving the Saudi Project for Assessment of Acute Coronary Syndrome (SPACE). (DOCX)

S1 Table Baseline characteristics prior to age and gender matching. (DOCX)

S2 Table Hospital therapies of study patients prior to age and gender matching. (DOCX)

S3 Table Adverse hospital outcomes prior to age and gender matching. (DOCX)
