# Supplementary material for: Disparities in Health Care Delivery and Hospital Outcomes between Non-Saudis and Saudi Nationals Presenting with Acute Coronary Syndromes in Saudi Arabia
Source: PLoS One. 2015 Apr 16;10(4):e0124012. doi: 10.1371/journal.pone.0124012 (PMC4399885; doi:10.1371/journal.pone.0124012)
Supplement: S1 Hospitals List — (DOCX) [file pone.0124012.s001.docx]

**The SPACE registry received ethics approval from the following ethics boards:**

1. King Khalid University Hospital institutional review board, Riyadh, KSA
2. Saud AlBabtain Cardiac Center Ethics board, Dammam, KSA
3. Security forces Hospital research and Ethics committee, Riyadh, KSA
4. King Faisal Specialist Hospital and Research Center institutional review board, Jeddah, KSA
5. King Khalid National Guard Hospital Ethics board, Jeddah, KSA
6. King Abdul Aziz University Hospital ethics board, Jeddah, KSA
7. King Fahd Medical City institutional review board, Riyadh, KSA
8. King Fahd Armed Forces Hospital ethics board, Jeddah , KSA
9. Prince Sultan Cardiac Center, Military Hospital ethics board, Riyadh, KSA
10. King Abdul-Aziz Medical City institutional review board, Riyadh, KSA
