# Supplementary material for: Disparities in Health Care Delivery and Hospital Outcomes between Non-Saudis and Saudi Nationals Presenting with Acute Coronary Syndromes in Saudi Arabia
Source: PLoS One. 2015 Apr 16;10(4):e0124012. doi: 10.1371/journal.pone.0124012 (PMC4399885; doi:10.1371/journal.pone.0124012)
Supplement: S1 Table — (DOCX) [file pone.0124012.s002.docx]

**S1 Table . Baseline characteristics of study patients with prior to age and gender matching**

|  | **Overall**  **n=5055**  **(%)** | **Saudi**  **n=4167**  **(82.4)** | **Non-Saudi**  **n=888**  **(17.6)** | ***p*-value** |
| --- | --- | --- | --- | --- |
| Age, y, mean ± SD | 58±12.9 | 59.7±12.8 | 50.2±10.5 | <0.0001 |
| Male, n (%) | 3915(77.4) | 3104(74.5) | 811(91.3) | <0.0001 |
| Diabetes mellitus, n (%) | 2937(58.2) | 2572(61.8) | 365(41.1) | <0.0001 |
| Hypertension, n (%) | 2782(55.3) | 2434(58.8) | 348(39.2) | <0.0001 |
| Hyperlipidemia, n (%) | 2082(41.4) | 1797(43.4) | 285(32.2) | <0.0001 |
| Smoking, n (%) | 2428(48.0) | 2043(49.0) | 385(43.3) | <0.0001 |
| CAD, n (%) | 716(14.2) | 570 (13.8) | 146(16.5) | 0.04 |
| Past PCI, n (%) | 700(13.9) | 624(15.0) | 76(8.6) | <0.0001 |
| CABG, n (%) | 296(5.9) | 258(6.2) | 38(4.3) | <0.0001 |
| CVA, n (%) | 309 (6.1) | 290 (7.0) | 19(2.1) | <0.0001 |
| PAD, n (%) | 202 (10.46%) | 170 (10.72%) | 32 (9.28%) | 0.545 |
| Unstable angina, n (%) | 1117 (22.1) | 987 (23.7) | 130 (14.6) | <0.0001 |
| STEMI, n (%) | 2099(41.5) | 1586(38.1) | 513(57.8) | <0.0001 |
| NSTEMI, n (%) | 1839 (3604) | 1594 (38.3) | 245 (27.6) | <0.0001 |
| Heart failure, n (%) | 776 (20.92) | 160 (19.12) | 936 (20.59) | 0.256 |
| Symptom-to-hospital arrival time, minutes, median (IQR) ^a^ | 143.5 ( 186.0 ) | 135.0 ( 180.0 ) | 161.5 ( 206.0 ) | 0.057 |
| HR < 100, n (%) | 677(14.8) | 558(15.0) | 119(14.1) | 0.3 |
| SBP ≤ 90, n (%) | 147 (3.2) | 116(3.1) | 31(3.7) | 0.2 |
| Waist circumference, n (%) | 99.80 ± 18.10 | 99.41 ± 17.83 | 100.9 ± 18.85 | 0.163 |
| Body mass index, n (%) | 28.20 ± 5.34 | 28.39 ± 5.42 | 27.38 ± 4.89 | <.001 |
| Total cholesterol, mean ± SD | 4.55 ± 2.32 | 4.47 ± 1.94 | 4.84 ± 1.39 | <.001 |
| LDL cholesterol, mean ± SD | 2.85 ± 2.73 | 2.75 ± 1.43 | 3.19 ± 3.63 | <.001 |
| FBS, mean ± SD | 7.68 ± 3.59 | 7.69 ± 3.28 | 7.50 ± 3.01 | 0.218 |
| Hemoglobin, mean ±SD | 13.87 ± 3.10 | 13.71 ± 2.97 | 14.53 ± 1.83 | <.001 |
| Creatinine, mean ± SD | 107.2 ± 78.98 | 108.1 ± 82.17 | 102.7 ± 61.18 | 0.063 |
| eGFR mean ± SD (ml/min/1.73 m²) | 74.33 ± 24.91 | 73.03 ± 25.19 | 80.43 ± 22.58 | <.001 |
| LV EF (< 35%), n (%) | 1713 (36.34) | 1467 (37.45) | 246 (30.87) | <.001 |
| Coronary angiography, n (%) | 3398(67.2) | 2914(70.0) | 484(54.5) | <0.0001 |
| LMS disease, n (%) | 161 (4.74) | 147 (5.04) | 14 (2.90) | 0.038 |
| 3-vessel disease, n (%) | 1145 (33.72) | 990 (33.99) | 155 (32.09) | 0.436 |
| 3-vessel or LM disease, n (%) | 1058 (36.31) | 163 (33.75) | 1221 (35.94) | 0.283 |

CABG, coronary artery bypass surgery; CAD, coronary artery disease; CVA, cerebrovascular accidents; eGFR, estimated glomerular filtration rate; FBS, fasting blood sugar; HR, heart rate; IQR, inter-quartile range; LMS, left main stem; LVEF, left ventricular ejection fraction; NSTEMI, non-ST elevation myocardial infarction; PAD, peripheral arterial disease; PCI, percutaneous coronary intervention; SBP, systolic blood pressure; SD, standard deviation; STEMI, ST elevation myocardial infarction.

^a^ Data from all STEMI patients
