# Supplementary material for: Disparities in Health Care Delivery and Hospital Outcomes between Non-Saudis and Saudi Nationals Presenting with Acute Coronary Syndromes in Saudi Arabia
Source: PLoS One. 2015 Apr 16;10(4):e0124012. doi: 10.1371/journal.pone.0124012 (PMC4399885; doi:10.1371/journal.pone.0124012)
Supplement: S2 Table — (DOCX) [file pone.0124012.s003.docx]

| **Variables** | **Overall**  **n=5055**  **(%)** | **Saudi**  **n=4167**  **(82.4)** | **Non-Saudi**  **n=888**  **(17.6)** | P-Value |
| --- | --- | --- | --- | --- |
| Aspirin n (%) | 4928 (97.7) | 4056 (97.5) | 872 (98.6) | 0.047 |
| Clopidogrel, n (%) | 4226 (83.8) | 3517 (84.6) | 709 (80.3) | 0.002 |
| β blockers, n (%) | 4117 (81.7) | 3388 (81.5) | 729 (82.6) | 0.2 |
| ACEI, n (%) | 3504 (69.5) | 2918 (70.2) | 586 (66.3) | 0.01 |
| ARBs, n (%) | 297 (5.92) | 265 (6.41) | 32 (3.63) | 0.001 |
| Statins, n (%) | 4704 (93.31) | 3872 (93.14) | 832 (94.12) | 0.335 |
| Fibrinolytic therapy^a^,n (%) | 627 (69.51) | 339 (60.86) | 288 (83.48) | <.001 |
| Primary PCI^a,^ n (%) | 158 (17.32) | 128 (22.65) | 30 (8.64) | <0.001 |
| Door-to-needle time, min, median (IQR)^a^ | 51.00 ( 54.00 ) | 60.00 ( 65.00 ) | 45.00( 41.50) | <.001 |
| Door-to-balloon-time, min, median (IQR)^a^ | 110.0 ( 68.00 ) | 119.5 ( 67.00 ) | 86.5( 23.00 ) | 0.001 |
| Door-to-needle-time < 30 min, n (%)^a^ | 144 (19.8) | 101 (19.8) | 43 (19.8) | 0.5 |
| Door-to-balloon-time < 90 min, n (%)^a^ | 46 (31.08%) | 30 (25.00%) | 16 (57.14%) | 0.003 |
| PCI, n (%) | 1775 (35.3) | 1509 (36.4) | 266 (30.0) | 0.001 |
| CABG, n (%) | 425 (8.5) | 353 (8.5) | 72 (8.1) | 0.5 |

**S2 Table. Hospital therapies of study patients prior to age and gender matching**

ACEI, angiotensin converting enzyme inhibitors; ARBs, angiotensin receptor blockers; CABG, coronary artery bypass surgery; IQR, inter-quartile range; PCI, percutaneous coronary intervention.

^a^ Data from all STEMI patients
