# Supplementary material for: Disparities in Health Care Delivery and Hospital Outcomes between Non-Saudis and Saudi Nationals Presenting with Acute Coronary Syndromes in Saudi Arabia
Source: PLoS One. 2015 Apr 16;10(4):e0124012. doi: 10.1371/journal.pone.0124012 (PMC4399885; doi:10.1371/journal.pone.0124012)
Supplement: S3 Table — (DOCX) [file pone.0124012.s004.docx]

| **Outcome**  **n (%)** | **All patients**  **n=5055** | **Saudi nationals**  **n=4167** | **Non-Saudis**  **n=888** | ***p*-value** |
| --- | --- | --- | --- | --- |
| Death | 155 (3.1) | 129 (3.1) | 26 (2.9) | 0.4 |
| Heart failure | 520 (10.3) | 449 (10.8) | 71 (8.0) | 0.007 |
| Stroke | 48 (1.0) | 40 (1.0) | 8 (0.9) | 0.5 |
| Re-infarction | 77 (1.5) | 67 (1.6) | 10 (1.1) | 0.2 |
| Cardiogenic shock | 221 (4.4) | 181 (4.3) | 40 (4.5) | 0.4 |
| Major bleeding | 68 (1.3) | 61 (1.5) | 7 (0.8) | 0.07 |

**S3 table. Adverse hospital outcomes prior to age and gender matching**
